# *Clostridium butyricum* maximizes growth while minimizing enzyme usage and ATP production: metabolic flux distribution of a strain cultured in glycerol

**DOI:** 10.1186/s12918-017-0434-0

**Published:** 2017-06-01

**Authors:** Luis Miguel Serrano-Bermúdez, Andrés Fernando González Barrios, Costas D. Maranas, Dolly Montoya

**Affiliations:** 1 Bioprocesses and Bioprospecting Group, Universidad Nacional de Colombia. Ciudad Universitaria, Carrera 30 No. 45-03, Bogotá, D.C Colombia; 2 Grupo de Diseño de Productos y Procesos (GDPP), Departamento de Ingeniería Química, Universidad de los Andes, Carrera 1 N.° 18A – 12, Bogotá, Colombia; 3 Department of Chemical Engineering, The Pennsylvania State University, University Park, PA 16802 USA

**Keywords:** *Clostridium butyricum*, 1,3-propanediol, Genome-scale metabolic model, Objective function

## Abstract

**Background:**

The increase in glycerol obtained as a byproduct of biodiesel has encouraged the production of new industrial products, such as 1,3-propanediol (PDO), using biotechnological transformation via bacteria like *Clostridium butyricum*. However, despite the increasing role of *Clostridium butyricum* as a bio-production platform, its metabolism remains poorly modeled.

**Results:**

We reconstructed *i*Cbu641, the first genome-scale metabolic (GSM) model of a PDO producer *Clostridium* strain, which included 641 genes, 365 enzymes, 891 reactions, and 701 metabolites. We found an enzyme expression prediction of nearly 84% after comparison of proteomic data with flux distribution estimation using flux balance analysis (FBA). The remaining 16% corresponded to enzymes directionally coupled to growth, according to flux coupling findings (FCF). The fermentation data validation also revealed different phenotype states that depended on culture media conditions; for example, *Clostridium* maximizes its biomass yield per enzyme usage under glycerol limitation. By contrast, under glycerol excess conditions, *Clostridium* grows sub-optimally, maximizing biomass yield while minimizing both enzyme usage and ATP production. We further evaluated perturbations in the GSM model through enzyme deletions and variations in biomass composition. The GSM predictions showed no significant increase in PDO production, suggesting a robustness to perturbations in the GSM model. We used the experimental results to predict that co-fermentation was a better alternative than *i*Cbu641 perturbations for improving PDO yields.

**Conclusions:**

The agreement between the predicted and experimental values allows the use of the GSM model constructed for the PDO-producing *Clostridium* strain to propose new scenarios for PDO production, such as dynamic simulations, thereby reducing the time and costs associated with experimentation.

**Electronic supplementary material:**

The online version of this article (doi:10.1186/s12918-017-0434-0) contains supplementary material, which is available to authorized users.

## Background

The rising biodiesel industry has resulted in a major overproduction of glycerol as a byproduct, which now threatens the economic viability of this industry [1, 2]. This situation has spurred research into glycerol utilization as a carbon source [3–5] and for the generation of products such as 1,3-propanediol (PDO), a precursor of important commercial polymers, such as polyester and polyurethane [6, 7]. PDO can be biosynthesized from glycerol by bacteria such as *Clostridium butyricum* or *Klebsiella* spp. [3, 7]. *Clostridium* species are the more attractive alternative because they are safer and achieve higher yields than *Klebsiella* [8]. However, industrial PDO production using bacteria is still limited by insufficient yields, which presents a serious obstacle to the competitiveness of this process [9–11]. Therefore, strategies such as fed-batch cultures and random mutagenesis have been developed, resulting in improvements in PDO production of up to 137% and 78%, respectively [11–13]. A more detailed understanding of the metabolic pathways in species such as *Clostridium butyricum* could therefore shed light on a better ways to promote glycerol transformation to PDO in this organism.

Metabolism studies of glycerol by the anaerobic bacterium *Clostridium butyricum* have generally focused on its central metabolism, which is composed of oxidative and reductive branches [14]. The oxidative branch is mainly related to the production of ATP and reducing equivalents (NADH), with the formation of acetic and butyric acids as byproducts. By contrast, the reductive branch produces PDO while simultaneously regenerating reducing equivalents by conversion of NADH to NAD [7, 9, 15]. Bizukojc et al. [16] reported the most detailed metabolic model for a PDO producer *Clostridium* strain, indicating the functioning of 77 reactions and 69 metabolites. The model, in addition to the oxidative and reductive branches, also included simplified synthesis reactions for amino acids, macromolecules, and biomass. However, at present, metabolic models based on genome annotation information, also known as genome-scale metabolic (GSM) models [17, 18], are lacking for *Clostridium butyricum*.

A proteomics study of the native Colombian strain *Clostridium* sp. IBUN 158B cultured in glycerol [19] has provided experimental validation of the enzyme expression involved in PDO metabolic networks in this specie. The proteome contained 21 enzymes classified as follows: one from the reductive branch (PDO dehydrogenase), three from the oxidative branch, eleven from carbohydrate synthesis, four from amino acid synthesis, and two from nucleotide synthesis. Gungormusler et al. [20] also used proteomics for the experimental detection of 262 different enzymes expressed by *Clostridium butyricum* 5521 cultured in glycerol. Nevertheless, despite this experimental information and the computational tools available, the prediction of PDO production by *Clostridium* based on its metabolic behavior is still limited.

One computational tool commonly employed for metabolic modeling is flux balance analysis (FBA). FBA allows the use of a steady state assumption of defined culture conditions to predict the phenotype of one microorganism based on its GSM model [21–25]. However, a GSM model expressed as stoichiometric matrix is an undetermined system, that is, it has more reactions than metabolites. This creates a situation with infinite solutions, so an objective function is required to predict the microorganism phenotype. FBA then becomes an optimization process in which the constraints are the culture conditions, mass balances, and thermodynamic feasibilities [22, 25–28].

In general, predictions using GSM models assume biomass yield maximization as the objective function, based on the assumption that cells have evolved to select the most efficient pathways that achieve the best yields [29]. Nevertheless, predictions with biomass maximization do not always capture the cellular physiology, and alternative objective functions have been developed [28, 30–33]. Studies have included error minimization by bi-level optimization [30, 34, 35], objective function selection by Bayesian inference [31] or by Euclidian distance minimization [32], and linear combination of objective functions [28, 36]. The results, overall, highlight that a cell does not maximize biomass yield under scenarios like substrate excess, so that one single function is unable to predict all the evaluated scenarios [28, 32, 33, 37–39].

For these reasons, the initial purpose of the present research was to construct the first GSM model of a PDO producer *Clostridium* strain. The biological model selected was the Colombian strain *Clostridium* sp. IBUN 13A, a strain isolated by our Bioprocesses and Bioprospecting Group. This strain is a natural PDO producer and has been employed over the last 20 years in several studies aimed at understanding PDO production, including the annotation of its genome [40–42]. Additionally, as second objective, our intent was to predict the phenotypic states of this bacterium during culture in glycerol and in other substrates using the GSM model and FBA with the adequate objective function. Our overall aim was to evaluate the effect of perturbations in the constructed GSM model on PDO yield improvements.

## Results and discussion

### Genome-scale metabolic model *i*Cbu641 reconstruction and curation

A draft metabolic model for the *Clostridium* sp. IBUN 13A strain was constructed based on RAST annotation [41]. The draft was composed of 641 genes, 365 enzymes, 671 reactions, and 606 metabolites. GapFind [43] analysis of the draft model identified 303 blocked metabolites, which were reduced to 63 by adding 59 reactions based on experimental fermentation evidence from *Clostridium butyricum* cultured in glycerol [15, 19, 44] and on curated GSM models from other solventogenic clostridia [16, 45–52]. The biomass reaction was adapted from *C. beijerinckii* GSM [45], which does not account for the proton formation associated with ATP hydrolysis during the growth-associated maintenance (GAM), as is also observed in *C. acetobutylicum* [49]. This excluded proton would accumulate in the biomass, thereby preventing stabilization of the biomass charge. By contrast, the GSM models of *C. thermocellum* [50], *C*. *ljungdahlii* [52], and *C. cellulolyticum* [51] include this proton production in their biomass reactions. Therefore, the proton formation was included in the present biomass reaction to resolve the inconsistency in the elemental composition of the biomass, as well as the charge balance. The elemental composition per C atom, calculated based only on stoichiometric consumption of precursors, was therefore CH_1.624_O_0.456_N_0.216_P_0.033_S_0.0047_.

**Fig. 1:**
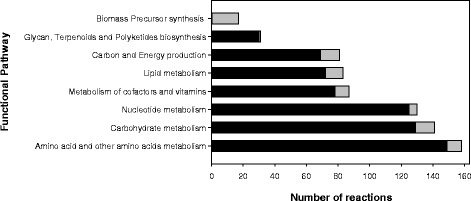
Distribution of cytosolic reactions in the *i*Cbu641 GSM model by functional pathway. Notation (■) Gene-Associated reactions (■) Non Gene-Associated reactions

After curation, elemental balancing, and loop deletion, the constructed *i*Cbu641 GSM model included 641 genes, 891 reactions, and 701 metabolites. Table 1 summarizes the main features of the curated metabolic network, and Fig. 1 shows the pathway distribution of the cytosolic reactions. Comparison with other GSM models of solventogenic *Clostridium* strains showed 11 unique enzymes, including as PDO dehydrogenase (EC.1.1.1.202) and glycerol dehydratase (EC.4.2.1.30). These two enzymes function in lipid metabolism but are associated with the reductive branch of glycerol metabolism [14].

**Table 1:** Main features of the *i*Cbu641 metabolic network

Feature		Number
Genes		641
Enzymes		365
Total Reactions		891
	Cytosolic reactions^a^	727
	Transport reactions	86
	Exchange reactions	78
Total Metabolites		701
	Blocked metabolites	63

^a^Includes 17 simplified biomass and macromolecule synthesis reactions [45] and 59 reactions added in the curation

The *i*Cbu641 metabolic network is the first GSM model curated for a PDO producing *Clostridium* strain [17, 18, 49] (See Additional files 1 and 2 for complete metabolic model at excel format and SBML format, respectively). The *i*Cbu641 model also includes all the enzymes associated with glycolysis and the pentose phosphate pathway and most of the enzymes involved in the TCA cycle. The enzymes from the TCA cycle that were not included are malate dehydrogenase (EC.1.1.5.4), succinate-CoA ligase (EC.6.2.1.4 - EC.6.2.1.5) and fumarate reductase (EC.1.3.5.4), which were not detected in the genome. Therefore, additional experimentation is required to verify the presence or absence of genes encoding these three enzymes in the genome of *Clostridium* sp. IBUN 13A. The model is able to synthesize de novo all the precursors involved in the biomass reaction (e.g., amino acids, nucleotides, fatty acids, teichoic acid, and cofactors).

### Flux distribution prediction using flux balance analysis with glycerol as substrate

**Fig. 2:**
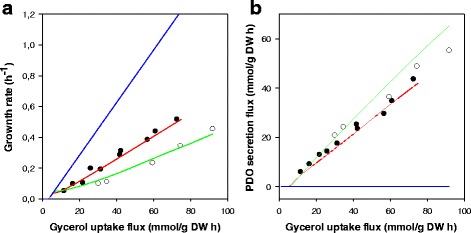
Robustness analyses in function of glycerol uptake flux. **a** Specific growth rate μ and **b** PDO secretion flux *v*_*PDO.*_ Notation: Experimental data from Solomon et al. [44] at (●) glycerol limitation and (○) glycerol excess. FBA predictions using: biomass maximization (Blue Line), biomass maximization per enzyme usage (Red Line) and biomass maximization while minimizing both enzyme usage and ATP production (Green Line)

The constructed *i*Cbu641 GSM model and FBA were employed to predict the flux distribution of *Clostridium butyricum* cultured in glycerol. FBA was solved using linear programming (LP) with biomass maximization as an objective function. When compared with the experimental data, FBA predicted a biomass overestimation and no PDO production (Fig. 2 – Blue lines), giving biomass yield (Y_X/S_) and PDO yield (Y_PDO/S_) errors of 300% and 100%, respectively [44]. Therefore, taking into account only biomass yield, we could infer that, experimentally, *Clostridium butyricum* does not grow optimally in glycerol. This can be explained mathematically, because the objective function and all constraints (mass balances and thermodynamic feasibilities) used were linear. This means that the optimum found is indeed a vertex of the feasible solution space [38], where no PDO could be produced.

Contrary to FBA predictions, PDO production in *Clostridium* sp. IBUN 13A is related to *dha* operon expression and the enzyme activity of both PDO dehydrogenase (EC.1.1.1.202) and glycerol dehydratase (EC.4.2.1.30), which have been experimentally detected in the presence of glycerol as main carbon source [40, 53, 54]. The lack in predictions can be interpreted biologically based on redox balance together with the capability of *C. butyricum* to produce formic acid and hydrogen (H_2_) [44]. *Clostridium butyricum* is an anaerobic bacterium, so according to the redox balance, the substrate must act simultaneously as an acceptor and donor of electrons [55]. However, the aforementioned capability allows to LP optimization predicts that the substrate could be used mostly as an electron donor, thereby generating more ATP and biomass. This prediction is achieved due to the regeneration of reducing equivalents through formic acid and H_2_ formation, which would require no substrate as an electron acceptor to produce reduced compounds such as PDO. In other words, in the vertex predicted by LP optimization, the substrate is used mainly as an electron donor due to the formation of formic acid and H_2_, and no PDO is produced.

Similar results were observed using glucose as a substrate, where formic acid and H_2_ were overproduced instead of reduced products like butanol or ethanol, which have been detected experimentally (data not shown) [56]. Consequently, LP optimization could be considered as unsuitable for predicting the experimental yields of *Clostridium butyricum*, which is capable of producing formic acid or H_2_. Nevertheless, LP optimization is commonly employed in solventogenic *Clostridium* strains, although all of them are able to produce at least hydrogen [45, 47, 48, 50, 51]. This lack of prediction could be solved using experimental constraints of formic acid and H_2_ secretion in LP optimization; however, no linear trend was observed, especially in H_2_ secretion [44].

A new objective function of maximizing biomass yield per flux unit (Equation 1) was therefore employed to improve the FBA predictions. This objective function is based on the hypothesis that cells operate to maximize biomass yield while minimizing enzyme usage [32]. This non-linear programming (NLP) optimization is non-convex, but Schuetz et al. suggested that the predicted local optimum is indeed the global optimum [32]. In addition to the new objective function, a non-linear constraint was employed, which corresponds to the maximum acetic acid secretion flux and has an allosteric trend in the function of glycerol uptake flux [44]. Therefore the constraint was not used during simulations with other carbon sources, as glucose. This allosteric trend of acetate production has been previously reported for *Clostridium butyricum* cultured in glycerol as a mechanism to control acetyl-CoA/CoA and ATP/ADP ratios [57]. The new simulations (Fig. 2 – Red lines) predicted Y_X/S_ and Y_PDO/S_ errors of nearly 4.5%, and 1.5%, respectively, when compared with the experimental values obtained under limiting conditions of glycerol (<15 g/L) [44]. Therefore, the error reduction using NLP predictions suggests the resolution of the redox balance problems observed in LP optimization caused by the capability of producing formic acid and H_2_. It also suggests that *Clostridium butyricum* indeed minimizes enzyme usage and prefers short pathways (PDO production) to maximize its growth under limiting nutrient conditions.

1 $$ \mathit{\operatorname{Max}}\ \frac{\mu}{\sum_{i=1}^n{v}^2} $$

Identical phenotypic predictions were also observed using both LP and NLP optimizations when the production of hydrogen and formic acid were blocked as additional constraints (data not shown). This validates, on the one hand, the effect of these products in the lack of prediction using LP optimization. On the other hand, NLP optimization indeed predicts global optima, as suggested Schuetz et al. [32].

However, the new objective function overestimated Y_X/S_ under glycerol-excess conditions (without reaching inhibition conditions) [44], suggesting that *Clostridium butyricum* grows under sub-optimal conditions when the substrate is present in excess, as is also observed in *E. coli* [32]. This behavior is understandable if thermodynamics is considered. Growth, as with any reaction, is thermodynamically feasible if its driving force, expressed as Gibbs free energy, is negative (ΔG_growth_ < 0). Growth Gibbs free energy can be expressed as a function of biomass yield and the Gibbs free energies for catabolism and anabolism, as shown Eq. 2 [55]. Therefore, an improvement in thermodynamic feasibility will be coupled to biomass yield reduction, as occurs in scenarios such as substrate competition with other microorganisms [55]. This suggests that a substrate excess condition could induce the above scenario in order for the organism to prevail in this environment.

2 $$ {\Delta G}_{growth}={\frac{1}{Y_{X/ S}}\Delta G}_{catabolism}+{\Delta G}_{anabolism} $$

The sub-optimal behavior was accounted for in the objective function of Eq. 1 by the use of a tunable weighting factor (*w*) that minimizes both the enzyme usage and ATP production of the network (Equation 3). The ATP incorporation in the objective function is based on experimental results [15, 44] and because a reduction in ATP production is related to the respective biomass yield reduction. Therefore, a weight *w* equal to 1 corresponds to the optimal conditions of Eq. 1. The use of weight factors has been described by Torres et al., who added ATP and NADH/NADPH minimization or maximization to the objective function, which improved *S. cerevisiae* growth predictions up to 98% [36, 39]. On this basis, simulations under excess conditions were made using a weight factor equal to 0.04 (*w* = 0.04), where the average experimental errors were minimal [44]. The average Y_X/S_ and Y_PDO/S_ errors were 5.3% and 2.5%, respectively, as shown Fig. 2 – Green Lines, confirming the ability of FBA to provide accurate predictions of these results through NLP optimization. However, this weight factor only applies to *Clostridium butyricum* cultured anaerobically in glycerol; additional experimental data would validate its usage.

3 $$ \mathit{\operatorname{Max}}\kern0.75em \frac{\mu}{w\left[\sum_{i=1}^n{v}^2\right]+\left(1- w\right)\left[{v_{ATP\  prod}}^2\right]}\kern.4em  w\in \left(0,1\right) $$

We further validated the necessity of employing the weight factor at sub-optimal conditions of glycerol by comparing both predicted phenotypes with the results reported by Zeng [15]. Zeng found that the directionality of the reaction catalyzed by ferredoxin reductase (EC.1.18.1.2 or EC.1.18.1.3) changes if glycerol is in limitation or in excess. Under the limiting condition, ferredoxin reductase consumes reducing equivalents and produces more H_2_ and less PDO than is observed under the excess condition, where ferredoxin reductase produces reducing equivalents. Consequently, Zeng quantified ferredoxin reductase directionality by calculating the ratio between hydrogen and reduced ferredoxin formed (${\alpha}_{H_2/ Fd}$). The experimental ratios are 1.1 and 0.4 under limiting and excess glycerol conditions, respectively. Similarly, the ratios predicted by FBA are 1.46 and 0.40 at optimal and sub-optimal conditions, respectively. Therefore, the predictions for both conditions agree with the reported values and validate the utility of the weight factor in predicting glycerol cultures.

We also compared the flux prediction for the 365 enzymes present in *i*Cbu641 with the experimental expression of the 286 enzymes detected by Gungormusler et al. in the proteome of the *Clostridium butyricum* DSM 10702 strain cultured under limiting glycerol conditions [20]. An analysis of the 174 common enzymes using flux couple finding (FCF) revealed 80 enzymes that were partially or fully coupled to growth and 67 enzymes that were directionally coupled to growth [58]. The remaining 27 enzymes were blocked and were therefore excluded from the comparison. These 27 enzymes could be blocked due to a lack of gene annotation or exclusion from biomass synthesis (e.g., terpenoid synthesis). Terpenoids have been detected in the cell walls of *Clostridium* strains and may arise as a stress response to the acids formed during culture [59]. The terpenoid pathway could be unblocked if these metabolites are added to biomass synthesis, but their concentration levels first need accurate quantification [59].

Assuming a qualitative correlation between the expression and flux for the 147 enzymes included in the comparison, FBA was able to predict the expression of 123 of them (83.7%). The remaining 24 non-predicted enzymes correspond only to directionally coupled growth and most of them (21 enzymes) are involved in carbohydrate metabolism and the synthesis of nitrogenous compounds (amino acids and nucleotides). The absence of a prediction for these enzymes could be due the presence of alternate pathways and isozymes, as is the case for asparagine synthase (EC.6.3.5.4), isocitrate dehydrogenase (NADP) (EC.1.1.1.42), and glycerol-3-phosphate dehydrogenase (EC.1.1.1.94), where the alternative enzymes are asparagine synthetase (EC.6.3.1.1), isocitrate dehydrogenase (NAD) (EC.1.1.1.41), and glycerol kinase (EC.2.7.1.30), respectively. Another possible cause is the inability of FBA to predict regulation mechanisms [60], as is the case for the enzyme pyruvate phosphate dikinase (EC.2.7.9.1), which is involved in the gluconeogenesis pathway but appears to act in the place of pyruvate kinase, consuming AMP instead ADP [61, 62]. Other enzymes, such as nicotinate phosphoribosyltransferase (EC.6.3.4.21) or pyrimidine nucleoside phosphorylase (EC.2.4.2.2), show a lack of prediction because they are involved in RNA or DNA fragment recycling [63]. Finally, 1,4-alpha-glucan branching enzyme (EC.2.4.1.18) or starch synthase (EC.2.4.1.21) were not predicted as they are part of granulose synthesis, a process that is not included in the biomass reaction. Granulose is a polysaccharide employed as carbon source during sporulation; therefore, it is produced during exponential growth [64]. (See Additional file 3 for complete proteomic comparison results).

### Flux distribution prediction using FBA and other carbon sources

The robustness of *i*Cbu641 was further tested by comparing the experimental and simulated data using other substrates at optimal conditions, Eq. 1. We first compared the FBA predictions and experimental yields of *Clostridium butyricum* W5 cultured in glucose [56], as shown in Table 2. We observed an accuracy of nearly 97% for predicting the biomass yield using this substrate, confirming the ability to predict not only glycerol cultures but also glucose cultures. All the reported experimental yields also agreed with their respective predicted feasible ranges calculated using flux variability analysis (FVA).

**Table 2:** Comparison of the experimental and simulated yields (mol/mol) of *Clostridium butyricum W5* cultured in glucose [56]

Product	Experimental Yield	Predicted feasible range
Biomass	0.0270	0.0279 (0.0228–0.0330)
Acetate	0.172	0.574 (0–1.053)
Lactate	0.566	0.179 (0–0.773)
Butyrate	0.295	0.114 (0–0.454)
H_2_	1.325	0.661 (0.046–1.345)
Ethanol	0.043	0.215 (0–0.714)

Junghare et al. [65] also evaluated biomass and hydrogen production of *Clostridium butyricum* TM-9A using different carbohydrates. Our comparison of the experimental yields with the predicted yields obtained through FBA is shown in Table 3. The trends in the predicted Y_X/S_ agree with the experimentally obtained values, showing smaller yields for pentoses and the highest yield for the trisaccharide raffinose, while the yields using monosaccharides were lower than those obtained using disaccharides. However, some differences are observed between the experimental and predicted values. First, ribose and xylose had considerably higher experimental than predicted yields. Second, the experimental yield for cellobiose was much lower than the predicted yield. Finally, although the simulations predicted the same yields for arabinose and ribose, their experimental yields differed. The first case could be a result of an incomplete curation of *i*Cbu641 related to pentose consumption; therefore, more experimental information is needed. The second and third cases could be due to miscalculation of the experimental yields of arabinose and cellobiose, since these substrates were not consumed completely, as can be inferred from their pH reports [65]. The last can point to the possibility that *Clostridium* was not well adapted to these substrates and may have needed to undergo more generations to reach its optimal growth [33]. The hydrogen yields (Y_H2/S_) showed experimental values that were mostly within their respective predicted feasible ranges. The only unpredicted Y_H2/S_ corresponded to arabinose, which supports the necessity of complementing *i*Cbu641 for consumption of pentoses. Consequently, *i*Cbu641 has the capacity to be employed to predict *Clostridium butyricum* growth using different carbohydrates as substrates.

**Table 3:** Comparison experimental and simulated yields (mol/mol) of *Clostridium butyricum* TM-9A cultured in different carbohydrates [65]

Carbohydrate	Experimental Yields		Predicted Yields	
	Y_X/S_^a^	Y_H2/S_	Y_X/S_^a^	Feasible range Y_H2/S_
Arabinose	2.5%	0.067	6.7%	0.204–1.101
Ribose	25.3%	0.843	6.7%	0.236–1.100
Xylose	32.3%	0.589	11.1%	0.327–1.406
Mannose	30.8%	0.668	36.2%	0.046–1.346
Fructose	32.2%	0.848	37.2%	0.092–1.374
Galactose	35.9%	0.864	29.1%	0.478–1.711
Cellobiose	35.9%	0.945	59.9%	0.141–2.468
Trehalose	65.7%	1.612	72.3%	0.030–2.485
Sucrose	74.9%	1.494	70.1%	0–2.323
Raffinose	100.0%	2.716	100.0%	0–3.141

^a^Biomass yields were normalized based on the raffinose value

We also assumed a qualitative correlation between the enzyme flux and mRNA expression and compared FBA predictions and experimental transcriptomics results for the *C. butyricum* strain CWBI 1009 cultured in glucose [66]. Of the 288 enzymes shared by both systems, 51 were blocked according FCF analysis and excluded from comparison. Among the remaining 237 enzymes, FBA predicted the activity of the 123 enzymes as partially and fully coupled to growth and 56 enzymes directionally coupled to growth, for a total of 179 predicted enzymes (75.5%). Similar to the proteomics comparison, the last 58 non-predicted enzymes were also directionally coupled to growth, and their lack of prediction is consistent with the reasons mentioned in the proteomics comparison. However, the lack of prediction of enzymes involved in thiamine production is highlighted, which is because this cofactor is not included as a biomass precursor. A similar situation happens with holo-ACP synthetase (EC.2.7.8.7), an enzyme involved in the CoA hydrolysis required to synthetize acyl carrier proteins (ACPs) [67]. (See Additional file 3 for complete transcriptomic comparison results).

Qualitative comparisons between proteomic and transcriptomic data require the assumption that enzymes are active, but this depends on different factors, like post-translational modifications, allosteric control, etc. [68]. Such factors are also a problem even when quantitative omics data are used in mathematical approaches that are employed to predict phenotype states [69]. However, qualitative proteomic and transcriptomic comparisons with FBA predictions have been previously reported using *E. coli* K12 by Lewis et al. [70], who found predictions for up to 82% of the evaluated enzymes, and most of the unpredicted ones were isozymes, in agreement with our results.

Finally, the *knockout* mutant of *Clostridium butyricum* W5 obtained by ClosTron technology [71] was employed to evaluate the ability of *i*Cbu641 to predict yields after perturbations in GSM. This mutant has butyric acid production blocked; its experimental yields are shown in Table 4. The wild type strain yields were predicted using FBA, while the mutant strain yields were obtained using regulatory on/off minimization (ROOM) [72]. ROOM was employed because it is better at predicting mutant phenotypic states when compared to other approaches, like minimization of metabolic adjustments (MOMA) [39]. This is because the ROOM approach seeks to maintain both the metabolic network and gene expression stabilities, as determined experimentally [72]. Simulations predicted an increased yield of ethanol using the mutant strain, and this was experimentally detected (Table 4). A biomass yield (Y_X/S_) reduction was also predicted for the mutant strain. By contrast, the experimental reduction of hydrogen yield in the mutant was not predicted; however, this is not conclusive since the FVA ranges of the wild type and mutant strains did agree with their experimental values.

**Table 4:** Comparison experimental and simulated yields of wild type and mutant strains of *Clostridium butyricum W5* cultured in glucose [71]

Product	Experimental yields		Simulated yields (FVA range)	
	Wild strain	Mutant strain	Wild strain (FBA)	Mutant strain (ROOM)
H_2_	1.25^a^	0.69	0.661 (0.046–1.345)	0.694 (0–1.787)
Ethanol	0.18	3.31 ^a^	0.215 (0–0.714)	0.680 (0–1.069)
Biomass^b^	100%	99.2%^a^	100%	95.0%

^a^Values calculated from information reported by Cai et al. [71]

^b^Yields reported as percentages based on the wild type strain

### Modeling scenarios with PDO yield increment

Three scenarios were evaluated to predict an increase in Y_PDO/S_ using *i*Cbu641. The first strategy was to use ROOM to predict single and double mutants through in silico enzyme deletion. The 145 enzymes associated with reactions directionally coupled to growth at culture conditions, as reported by Comba et al. [19], were considered in the analysis. Enzymes fully or partially coupled to growth were not considered, since their deletions would affect the culture time and therefore increase the fermentation costs. A total of 18 enzymes catalyzed at least two reactions that could block growth if simultaneously deleted, including dihydrodipicolinate reductase (EC.1.17.1.8) or proline oxidase (EC.1.5.1.2). Similar results were obtained for the 22 double enzyme deletions, such prephenate dehydrogenase (EC.1.3.1.12) and prephenate dehydrogenase (NADP) (EC.1.3.1.13) or shikimate dehydrogenase (EC.1.1.1.25) and quinate/shikimate dehydrogenase (EC.1.1.1.282). Most of the single and double deletions enhanced the Y_PDO/S_ by up to 1% in relation to the wild type strain. One of the best mutant predicted had simultaneous deletion of lactate dehydrogenases (EC.1.1.1.27 and EC.1.1.1.28), which increased Y_PDO/S_ only to nearly 1.2% (See Additional file 4 for complete mutant prediction results).

Single *knockout* mutants obtained from Colombian strain *Clostridium* sp. IBUN 158B [73] to improve the PDO production were used for validation. This is a different PDO producer strain, but research shows that the native strains currently sequenced share at least 99% of the genome (Article in preparation). Therefore, no significant differences were expected between the metabolic models of 13A and 158B, supporting the use of these mutants in validation. The inactivated enzymes were hydrogenase (Δ*hydA*-420 s), lactate dehydrogenase (Δ*ldhA*-508 s), and 3-hydroxybutyryl-CoA dehydrogenase (Δ*hbd*-414 s), corresponding the lack of production of hydrogen, lactic acid, and butyric acid, respectively. Montoya [73] reported that these three mutants were viable, as shown Table 5; however, he cultured only two of them due lack of time during his doctoral research. The biomass yield predictions for the mutants were overestimated, although a trend was predicted. This overestimation could be due to smaller glycerol uptake fluxes for the mutants, which would result in inadequate biomass yield normalization; however, the lack of measurements limits the elaboration of better comparisons.

**Table 5:** Comparison experimental and simulated yields of wild type and mutant strains of *Clostridium* sp. IBUN 158B cultured in glycerol [73]

Strain	Experimental yields		Simulated yields (FVA range)	
	Y_X/S_^a^	Y_PDO/S_	Y_X/S_^a^	Y_PDO/S_
Wild strain	100.0 ± 8%	0.538 ± 0.047	100%	0.588 (0.479–0.656)
Δ*hydA*-420 s	46.7 ± 9%	0.465 ± 0.031	75.8%	0.610 (0.478–0.721)
Δ*ldhA*-508 s	61.6 ± 3%	0.579 ± 0.021	98.9%	0.595 (0.489–0.659)
Δ*hbd*-414 s	Not available ^b^		95.2%	0.572 (0.448–0.656)

^a^Biomass Yields were normalized based on the wild type strain value

^b^Data not measured experimentally by Montoya [73]

The experimental values for PDO yields agreed with the predicted range, validating the ROOM simulations. Moreover, the FVA of the wild type strain indicated a PDO maximum flux that was 11.6% higher than the flux predicted by FBA; therefore, none of the mutants would show an increase in the PDO yield greater than this value without affecting biomass yield. This validates the single and double mutant predictions and suggests that mutant elaboration by *knockout* is an inadequate strategy for improving PDO yields. Similar results were obtained using *Optknock,* with up to 3 deletions [35, 74], where the maximum PDO yield predicted was 0.712 deleting hydrogen and butanol production. This agreed with the ROOM predictions shown in Table 5 and indicated a biomass yield reduction of nearly 28%. The *Optknock* maximum PDO yield value was 17.4% higher than the predicted value under glycerol limitation, but it was only 0.4% higher than the predicted value under glycerol excess, which reinforces that blocking reactions are useless. This can be understood by considering that PDO is a primary metabolite and its production is associated with growth [10, 75].

**Fig. 3:**
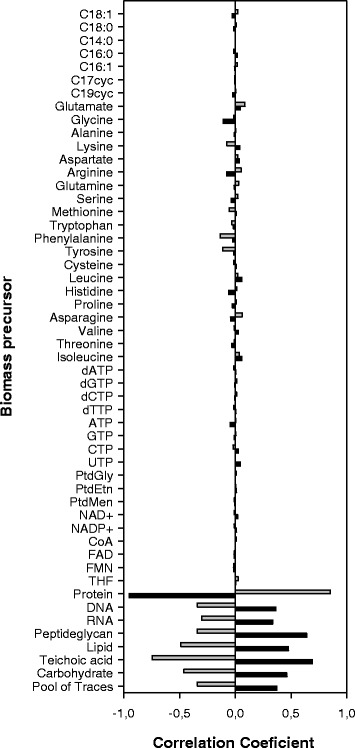
Pearson correlation coefficients of biomass and PDO yields to biomass precursors. Notation: (■) Y_X/S_ correlation. (■) Y_PDO/S_ correlation. Pearson values were calculated using a normalized random distribution of biomass precursors; a relative standard distribution of 30% was employed in all precursors. The covariance analyses were made with 9035 random combinations of precursor compositions

The second strategy evaluated was perturbation in the biomass composition by simultaneous random variation of stoichiometric coefficients of 44 precursors and 8 macromolecules. This strategy was evaluated since modifications in the culture media can affect biomass composition, such as accumulation of lipids during nitrogen starvation or protein accumulation with excess of nitrogen in the culture medium [76]. Normal distributions with relative standard deviations of 30% were employed for all stoichiometric coefficients considered in the perturbation. Figure 3 shows the Y_X/S_ and Y_PDO/S_ correlation values, indicating low correlation with the precursors (fatty acids, amino acids, nucleotides, polar lipids and cofactors) but a higher correlation with macromolecules, especially proteins (the main biomass component, accounting for 86.7% on a molar basis). This could suggest that a low protein content in the cell could improve Y_X/S_ and reduce the Y_PDO/S_, due their negative and positive correlations, respectively. However, the relative standard deviations obtained for Y_X/S_ and Y_PDO/S_ were 3.2% and 0.45%, respectively, meaning that the model is sufficiently stable to perturbations in biomass composition. These predictions suggest that changes in culture media aimed at modifying biomass composition and enhancing PDO yields would be unnecessary. However, modifications in the culture media could reduce PDO yield, as Dabrock et al. found using iron in excess [77].

**Fig. 4:**
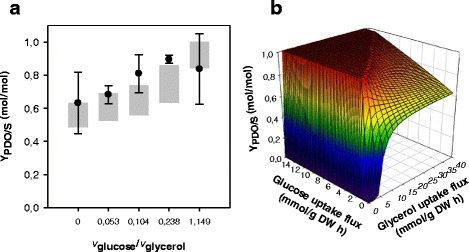
PDO yields using glucose and glycerol co-fermentation. **a** Comparison of experimental (scatter dots with standard deviation as error bars [78]) and FVA range prediction (Vertical boxes) of Y_PDO/S_ in the function of the glucose/glycerol uptake flux ratio. **b** Maximum Y_PDO/S_ predicted using FVA at different glucose and glycerol uptake fluxes. A ratio of glucose/glycerol uptake fluxes greater than or equal to 0.375 allows a complete conversion of glycerol to PDO (Y_PDO/S_ = 1). By contrast, PDO production is 0 without glycerol uptake flux (Y_PDO/S_ = 0)

The third strategy evaluated was to use two substrates simultaneously: glucose and glycerol. Glucose is used as carbon source, while glycerol is used to maintain redox balance, therefore generating higher Y_PDO/S_ values than obtained using glycerol as single substrate [78]. Figure 4a shows the experimental values and FVA ranges, which suggest that the cell operates at optimal conditions to produce PDO when glucose is present in the medium. This validation allowed an evaluation of the Y_PDO/S_ at different glucose and glycerol uptake fluxes, as shown Fig. 4b, which shows no PDO production (Y_PDO/S_ = 0) in the absence of a glycerol uptake flux. The results also show the complete transformation of glycerol to PDO (Y_PDO/S_ = 1), using ratios of at least 0.375 between uptake fluxes of glucose and glycerol. Therefore, these predictions permit the proposal that co-fermentation is the best alternative for improving biomass and PDO yields. However, a priori prediction of the molar ratio between these substrates that could allow these flux ratios is difficult.

## Conclusions

We generated *i*Cbu641, the first curated genome-scale metabolic model for a PDO producer *Clostridium* strain. During *i*Cbu641 validation, we solved flux balance analysis using LP optimization; however, according to the experimental data, the model predicted errors of nearly 300% for biomass yield and failed to predict PDO production. Therefore, NLP optimization was employed in FBA simulations, and the new objective function maximized biomass yield per flux unit [32]. The validation allowed prediction of appropriate growth and PDO production of cultures under glycerol limitation, but it still overestimated the experimental yields of cultures under glycerol excess. Thus, sub-optimal growth predictions under glycerol excess were achieved through a second NLP optimization, where ATP minimization was added to objective function. Therefore, both objective functions were able to predict *Clostridium butyricum* growth and PDO production under limiting and excess glycerol conditions. Additional validations were developed using proteomics and transcriptomics data, as well data from *knockout* mutants, which allowed verification of the accuracy of predicting perturbations of *i*Cbu641. All validations were completed using experimental data from different *Clostridium butyricum* strains and suggested that *i*Cbu641 is an agnostic GSM model at the state steady, but the differences may be observed during dynamic predictions.

Subsequently, perturbations in the metabolic network and biomass composition were proposed to increase the PDO yield predictions. However, these perturbations predicted no significant increments. We also evaluated glucose-glycerol co-fermentation as a strategy to improve PDO yields. We found that a ratio of glucose and glycerol uptake fluxes greater than or equal to 0.375 would allow the complete conversion of glycerol to PDO; however, experimental analysis is needed to find the molar ratio that allows the achievement of this flux ratio. Finally, predictions of PDO production in state steady cultures using *i*Cbu641 allows the proposal of this GSM model for predicting dynamic cultures (i.e. batch and fed-batch fermentations) capable of increasing PDO production, thereby minimizing the need for direct experimental efforts.

## Methods

### Genomic scale metabolic model *i*Cbu641 reconstruction

The draft genome was obtained for the Colombian-native strain *Clostridium* sp. IBUN 13A, isolated and stored by the Bioprocesses and Bioprospecting Research Group from the Institute of Biotechnology of the Universidad Nacional de Colombia. This draft genome was previously sequenced and annotated and is available in GenBank with the accession no NZ_JZWG00000000.1 [41]. The strategy used for initial manual curation was reverse engineering proposed by Senger and Papoutsakis [47]; automated curation was also employed using GapFind and GapFill [43]. The GSM models of the solventogenic *Clostridium* strains *C. acetobutylicum* [46–49], *C. thermocellum* ATCC 27405 [50], *C. beijerinckii* NCIMB 8052 [45], *C*. *ljungdahlii* ATCC 55383 [52], and *C. cellulolyticum H10* [51] were used as a database for the curation. The resulting network is based on KEGG nomenclature, whereas the SEED database [79] was used in mass and charge balances at pH 7. Finally, thermodynamically infeasible loops were eliminated according to the methodology of Schellenberger et al. [80].

The values for growth-associated maintenance (GAM) and non-growth–associated maintenance (NGAM) were reported for *Clostridium acetobutylicum* ATCC 824 by Lee et al. and are 40 mmol·ATP·g^−1^ and 5 mmol·ATP·g^−1^·h^−1^, respectively [46]. An allosteric model was also included as an upper bound constraint for the acetic acid secretion flux in the function of glycerol uptake flux. This trend was obtained using the experimental data reported by Solomon et al. [44] and Papanikolaou et al. [81]. The kinetic model was expressed as a logistic function, with 0.158 and 11.5·mmol·g^−1^ h^−1^ as initial and maximum values, respectively, and −0.0879·g·h·mmol^−1^ as the accumulation rate, as shown in Eq. 4.

4 $$ {v}_{acetic\  acid}\le \kern.75em \frac{11.5^{\ast }{0.158}^{\ast }{e}^{\left(-{0.0859}^{\ast }{v}_{glycerol}\right)}}{11.5+{0.158}^{\ast}\left({e}^{\left(-{0.0859}^{\ast }{v}_{glycerol}\right)}-1\right)} $$

### Flux balance analysis

The dynamics of the mass balance of metabolite *x*_*i*_ involved in *N* reactions is described in Eq. 5, where *S*_*ij*_ is the stoichiometric coefficient of metabolite *i* in reaction *j,* and *v*_*j*_ is the flux value in which this reaction occurs [25, 80]. Now, assuming a steady state, Eq. 5 can be expressed for *M* metabolites; however, since *N* > *M,* the prediction of fluxes *v*_*j*_ can be achieved using FBA, which maximizes or minimizes an objective function *Z* (Equation 6). The constraints of this function are the mass balances for the *M* metabolites and the upper *v*_*j*_^*max*^, and lower *v*_*j*_^*min*^ bounds of the N fluxes *v*_*j*_ [28, 31]. Additionally, feasible ranges of fluxes predicted by FBA are calculated using FVA. Since the objective functions employed are non-linear, the objective function value *Z* calculated with FBA has to be relaxed by 5%, as suggested Mahadevan et al., Eq. 7 [82]. The mutant phenotypes were predicted using the ROOM approach, Eq. 8, where *b*_*j*_ is the binary number of reaction *j* [72]. Also, *v* ^*l*^_*j,wild*_ and *v* ^*u*^_*j,wild*_ are the lower and upper confidence limits of wild type flux *j*. δ and ε are relative and absolute tolerance ranges, respectively.

5 $$ \frac{d{ x}_i}{ d t}=\sum_{j=1}^N{S}_{i j}{v}_i $$

6 $$ \begin{array}{c}\mathit{\operatorname{Max}}/\mathit{\operatorname{Min}}\kern1.25em  Z= f\left({v}_j\right)\kern9.25em \\ {}\begin{array}{c} Subject\  to\kern13.75em \\ {}\sum_{j=1}^N{S}_{ij}{v}_j=0,\kern.75em \forall i\in 1,\dots, M\\ {}{v}_j^{min}\le {v}_j\le {v}_j^{max},\kern.75em \forall j\in 1,\dots, N\end{array}\end{array} $$

7 $$ \begin{array}{c}\begin{array}{c}\mathit{\operatorname{Max}}/\mathit{\operatorname{Min}}\kern1.25em {v}_j\kern15em \\ {} Subject\  to\kern15.75em \\ {}\sum_{j=1}^N{S}_{ij}{v}_j=0,\kern.3em \forall i\in 1,\dots, M\kern2.75em \end{array}\\ {}{v}_j^{min}\le {v}_j\le {v}_j^{max},\kern1.25em \forall j\in 1,\dots, N\kern.5em \\ {}{Z}_{relaxed}=\left\{\begin{array}{c}1.05 Z\kern0.5em  if\  Z\  was\  minimized\\ {}0.95 Z\kern0.5em  if\  Z\  was\  maximized\end{array}\right.\end{array} $$

8 $$ \begin{aligned} &\text{Min} \sum^{N}_{j=1}b_j \\ &\ \ Subject\ to \\ &\quad\sum^{N}_{j=1} S_{ij}v_{j,mutant}= 0, \quad \forall i\in 1, \ldots, M\  \\ & \quad v_{j,mutant}^{max}\Lambda v_{j,mutant}^{min}\left\{ \begin{array}{llll} 0 & \forall & v_j & catalyzed\ only\ by\ enzyme\ ec\\ v_{j,wild}^{max} \Lambda v_{j,wild}^{min} & \forall & v_j & \left\{ \begin{array}{l} not\ cat\ by\ enzyme\ ec\\ cat\ by\ isozyme\ ofec \end{array}\right. \end{array}\right. \\ & \quad v_{j,mutant}-b_j\left( v_{j,mutant}^{max} -v_{j,wild}^{u}\right)\leq v_{j,wild}^{u} \quad \forall\quad j\in1,\ldots, N\\ & \quad v_{j,mutant}-b_j\left( v_{j,mutant}^{min} -v_{j,wild}^{l}\right)\geq v_{j,wild}^{l} \quad \forall\quad j\in1,\ldots, N\\ & \quad v_{j,wild}^{u} =v_{j,wild}+ \delta \left|v_{j,wild}\right|+\varepsilon \qquad \forall\quad j\in1,\ldots, N\\ & \quad v_{j,wild}^{l} =v_{j,wild}- \delta \left|v_{j,wild}\right|-\varepsilon \qquad \forall\quad  j\in1,\ldots, N\\ & \quad b_{j} = [0,1] \quad \forall\quad  j\in1,\ldots, N \\ & \quad \delta\approx0.05\qquad \varepsilon \approx 0.001\\ & \quad Subject\ to\\ & \qquad \ FBA\ of\ wild\ type\ strain\\ \end{aligned} $$

### Experimental validation

The experimental validation used two robustness analyses, as reported by Price et al. [83]: the former for the growth rate (μ) and the latter for PDO secretion flux (*v*_*PDO*_), both according to the glycerol uptake flux (*v*_*Glycerol*_). The robustness analyses were made using different objective functions for both glycerol limited and glycerol excess conditions. The objective function employed under glycerol limitation was biomass maximization per enzyme usage. Under glycerol excess, the biomass was maximized, while both enzyme usage and ATP production were minimized, where ATP production corresponds only to thermodynamically feasible reactions able to produce ATP. According to KEGG nomenclature, these reactions are R00156, R00158, R00200, R00315, R00332, R00512, R00570, R00722, R01512, R01547, R01665, R01688, R01724, R02090, R02093, R02094, R02098, R02326, R02331, R03005, R03035, R03530, and R03920. The predicted values were compared with the experimental values reported for the *Clostridium butyricum* DSM 5431 strain cultured in glycerol limited and glycerol excess conditions [44].

The prediction capability of enzyme expression was also evaluated by comparing the enzymes present in both metabolic model *i*Cbu641 and the experimental proteome of the strain *Clostridium butyricum* DSM 10702 cultured in glycerol [20]*.* The enzymes in the model were classified as blocked or directionally, partially or fully coupled to growth using FCF, according to the methodology reported by Burgard et al. [58]. Blocked enzymes were excluded from the comparison; this comparison assumed that all the expressed enzymes were active and catalyzed some reaction. Therefore, the enzyme expression could be: a) predicted when the flux of some of the reactions catalyzed by such enzyme is different to zero; b) not predicted when all the fluxes of reactions catalyzed by the enzyme expressed in the proteome are equal to zero.

Validation was also obtained using data from experimental cultures of *Clostridium butyricum* strains in substrates other than glycerol, such as glucose [56] and other carbohydrates [65]. The transcriptome reported by Calusinska et al. [66] for strain *C. butyricum* CWBI 1009, was also used for validation; this organism had been cultured in glucose using batch fermentation with uncontrolled pH. The mRNA detected in the transcriptome coded a total of 913 enzymes (532 unique and 381 redundant), where values at the exponential growth phase were used in a similar way to those from the proteomic data.

### In silico perturbations of the metabolic model

Regulatory on/off minimization (ROOM) [72] was employed as a strategy for prediction of the phenotypic states of mutants by *knockout* of enzymes directionally coupled to growth. In total, 145 single mutants were evaluated. Double deletion was also studied by simultaneously blocking two enzymes, excluding enzymes previously detected as essential in the single deletion; this led to evaluation of almost 7900 double mutants.

We also made perturbations in the biomass composition expressed by the variation of stoichiometric coefficients of 8 macromolecules, 7 fatty acids, 20 amino acids, 8 nucleotides, 3 polar lipids, and 6 cofactors. FBA was performed using different compositions randomly generated using normal distributions with standard deviations equivalent to 30% the values used in GSM model *i*Cbu641. A total number of 10,000 simulations were made, where 965 of them were excluded because at least one of the concentrations was negative. Coefficients of correlation were calculated for biomass and PDO yields using the remaining 9035 biomass precursor combinations.

### Technical implementation

FBA, FVA, and ROOM were computer simulated using GAMS (General Algebraic Modeling System, GAMS Development Corp., Washington, DC) software V.24.2.2 r44857 for Linux. Linear and Nonlinear Programming (LP and NLP) were developed with solver CONOPT v3.15 N, and Mixed Integer Programming (MIP) was developed with solver CPLEX 12.6.0.0. Data were analyzed using Microsoft Excel® 2010.

## Additional files

Additional file 1: Excel format of *i*Cbu641Genome Scale Metabolic Model. (XLSX 203 kb)

Additional file 2: SBML format of *i*Cbu641Genome Scale Metabolic Model. (XML 2250 kb)

Additional file 3: Proteomic and transcriptomic comparison results. (XLSX 30 kb)

Additional file 4: Results of modeling scenarios with PDO yield increment. (XLSX 5507 kb)

## Abbreviations

ACP: Acyl carrier proteins
FBA: Flux balance analysis
FCF: Flux couple finding
FVA: Flux variability analysis
GAM: Growth-associated maintenance
GSM: Genome-scale metabolic
LP: Linear programming
MIP: Mixed integer programming
MOMA: Minimization of metabolic adjustments
NGAM: Non-growth-associated maintenance
NLP: Non-linear programing
PDO: 1,3-Propanediol
ROOM: Regulatory On/Off Minimization
